# Cholinesterase-like organocatalysis by imidazole and imidazole-bearing molecules

**DOI:** 10.1038/srep45760

**Published:** 2017-04-03

**Authors:** Paola Nieri, Sara Carpi, Stefano Fogli, Beatrice Polini, Maria Cristina Breschi, Adriano Podestà

**Affiliations:** 1 Department of Pharmacy, University of Pisa, Pisa, Italy; 2 Department of Veterinary Sciences, University of Pisa, Italy

## Abstract

Organocatalysis, which is mostly explored for its new potential industrial applications, also represents a chemical event involved in endogenous processes. In the present study, we provide the first evidence that imidazole and imidazole derivatives have cholinesterase-like properties since they can accelerate the hydrolysis of acetylthiocholine and propionylthiocholine in a concentration-dependent manner. The natural imidazole-containing molecules as L-histidine and histamine show a catalytic activity, comparable to that of imidazole itself, whereas synthetic molecules, as cimetidine and clonidine, were less active. In the experimental conditions used, the reaction progress curves were sigmoidal and the rational of such unexpected behavior as well as the mechanism of catalysis is discussed. Although indirectly, findings of the present study suggests that imidazolic compounds may interfere with the homeostasis of the cholinergic system *in vivo*.

Binding specificity is the cornerstone event that characterizes the vast majority of structural and kinetic properties of the living state of matter. Conformational flexibility and chemistry of heteroatom substituents allow enzymes to efficiently catalyze specific chemical reactions in a stereospecific way. Nonetheless, many non-enzymatic reactions occur in living organisms including non-specific protein glycosylation^1^, reduction of sugars with free amino groups in proteins, lipids and nucleic acids^2^, lipid peroxidation by free radicals^3^, and hemoglobin degradation by t-butyl hydroperoxide^4^.

Until the discovery of organocatalysis in the late 1990s, the field of asymmetric catalysis (dedicated to the synthesis of chiral molecules) was dominated by the use of organometallic compounds and enzymes. Since then, more than one hundred of reaction types have been described in which monomeric organic molecules were able to promote enantioselective catalysis *in vitro*^5^,^6^,^7^,^8^,^9^, even in an aqueous environment^10^,^11^. This evidence suggests that, under appropriate circumstances, some small molecules may act as catalysts also *in vivo* influencing specific biological processes. Experimental confirmation of such a hypothesis may represent the rational base to synthesize new drug candidates^12^ to be used in pathological conditions characterized by the loss of endogenous enzyme activity.

The imidazole moiety of the amino acid histidine plays a role in the mechanism of catalysis of a number of hydrolytic enzymes, including trypsin, chymotrypsin, and acetylcholinesterase. The catalytic role of the imidazole group linked to polymers other than proteins^13^ or as part of small molecules^14^ has been also reported. Imidazole may function *per se* as a catalyst by promoting hydrolysis of phenyl acetate^15^, N-acetylserinamide and p-nitrophenol esters^16^, and cyclic peptides^17^, or accelerating the *in vitro* rate of glycerolipids synthesis^18^ and the transfer of activated acyl groups^19^. Furthermore, several imidazole analogues or derivatives have attracted the interest of the scientific community for their organocatalytic properties^20^.

In the present study, we provide the first evidence that imidazole and other endogenous or synthetic imidazole-containing molecules have a concentration-dependent cholinesterase-like activity and discuss possible physiological and pharmacological implications.

## Results

**Figure 1:**
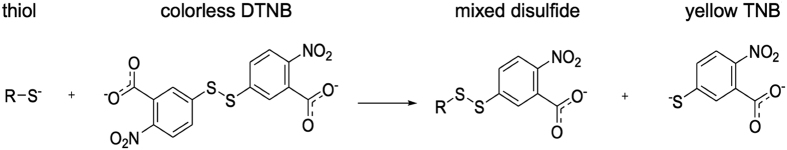
Reaction of the Ellman’s reagent (DTNB) with a thiol.

Ellman’s assay is used to estimate free thiol groups. This reaction is based on the ability of the reagent 5,5′-dithiobis-(2-nitrobenzoic acid) (DTNB) to react with the thiol to give the mixed disulfide and 2-nitro-5-thiobenzoate (TNB). In water (at neutral and alkaline pH conditions) TNB is in its anionic yellow form TNB^2−^, which is quantified by measuring the absorbance at 405 nm (Fig. 1). Ellman’s assay for assessing cholinesterase activity is based on the use of thioester analogs of natural enzyme substrates, i.e. acetylthiocholine (ACTh), propionylthiocholine (PTCh) or butirrylthiocholine (BTCh)^21^.

For the correct assessment of the catalytic activity of imidazole and imidazole derivatives, we measured the extent of thioester hydrolysis that spontaneously occurs when thioesters are dissolved in water. Since both electrophiles and, especially, nucleophile groups (such as the N-3 Lewis base) can cleave the S–S bond acting as polar reagents, we also evaluated this further source of blank hydrolysis. Findings of the present study showed that, in mild alkaline condition (pH 7.4), 40 mM imidazole caused a slight but significant increase in TNB^2−^ formation, even in the absence of thioester molecules in the solution. Results reported below were obtained by subtracting the absorbance due to blank hydrolysis.

**Figure 2:**
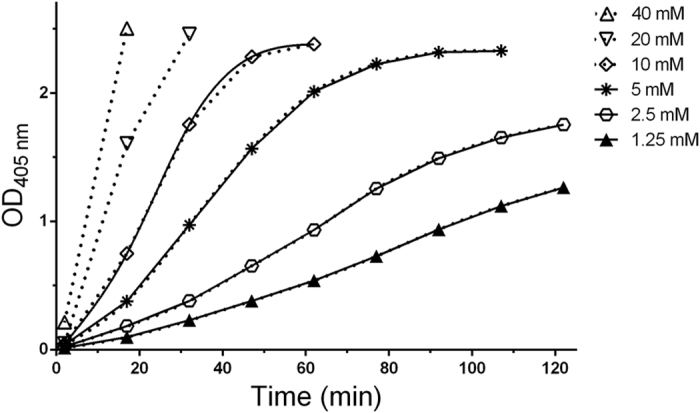
Concentration dependent cholinesterase-like activity of imidazole. Progress curves of TNB^2−^ formation (pH 7.4, T 37 °C) in the presence of a fixed [ATCh] (1 mM) and increasing [imidazole]. The solid line represents the cubic spline transform of the data series.

Figure 2 shows the progress curves of ATCh (1 mM) hydrolysis at 37 °C and pH 7.4, in the presence of increasing imidazole concentrations. Imidazole at 1.25–40 mM enhanced ATCh hydrolysis in a concentration-dependent manner. Progress curves are sigmoid and they represent true and continuous functions, as revealed by the superimposed cubic spline.

**Figure 3:**
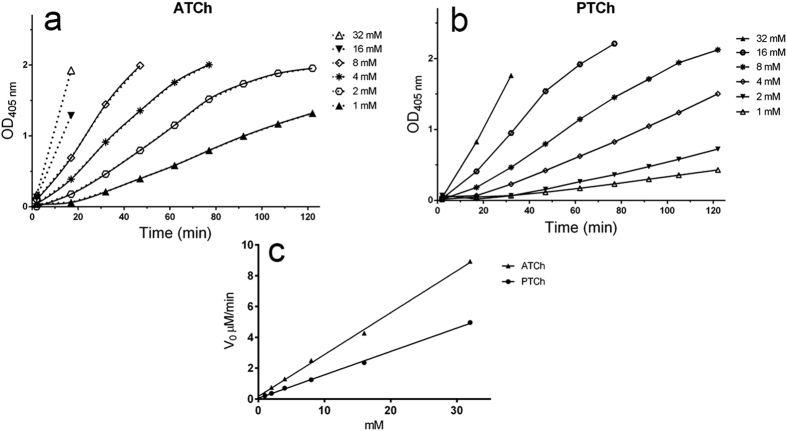
Imidazole organocatalytic activity vs different concentrations of Acetylthiocholine (ATCh) or Propionylthiocholine (PTCh). Progress curves of TNB^2−^ formation (pH 7.4, T 37 °C) in the presence of a fixed [imidazole] (1 mM) and increasing [ATCh] (**a**) or [PTCh] (**b**). The solid lines in panel **c** represents the cubic spline transforms of the data series. They are omitted in panel **b**. Linear correlations between apparent *v*_0_ and [ATCh] or [PTCh] are shown in panel **c**. See discussion for the rational of *v*_0(app)_ estimation.

Concentration dependency and sigmoidal behavior of dose-response curves were also observed in experiments performed at 1 mM imidazole (fixed concentration) in the presence of different ATCh or PTCh concentrations (Fig. 3a and b respectively). Apparent *k*_*cat*_ values were 4.53·10^−5^ ± 9.88·10^−7^ sec^−1^ for ATCh and 2.53·10^−5^ ± 4.40·10^−7^ sec^−1^ for PTCh. Plot of the apparent *v*_0_ vs. [S] is shown in panel c. Some data series were truncated to avoid the misleading effect caused by spontaneous hydrolysis of the thioester that increased proportionally with increasing ATCh or PTCh concentrations up to the limit to hide the catalyzed reaction.

**Figure 4:**
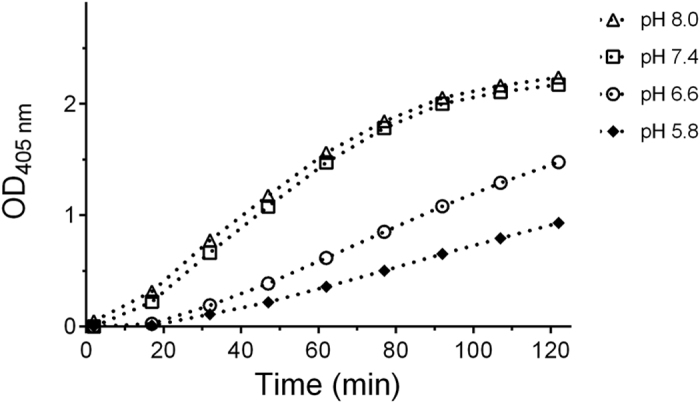
pH influence on imidazole organocatalytic activity. Progress curves of TNB^2−^ formation at different pH values (T = 37 °C, 1 mM ATCh, 5 mM imidazole).

**Figure 5:**
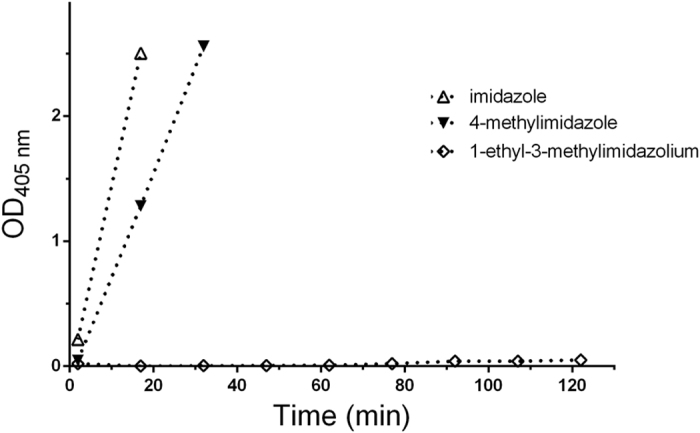
Comparison of 1-ethyl-3-methylimidazolium and 4-methylimidazole vs. imidazole activity. Progress curves of TNB^2−^ formation (pH 7.4, T 37 °C) in the presence of 1 mM ATCh and 40 mM imidazole, 1-ethyl-3- methylimidazolium chloride or 4-methylimidazole.

As shown in Fig. 4, imidazole catalysis was affected by the pH of the reaction mixture and catalytic efficiency increased as the solution became alkaline. This evidence suggested a role of the imidazole N-3 lone pair in the catalytic process. Such a hypothesis was further supported by data of Fig. 5 showing the trend of the hydrolysis curves obtained with 1 mM ATCh at pH 7.4 in the presence of 40 mM imidazole, 4-methylimidazole or 1-ethyl-3-methylimidazolium chloride.

**Figure 6:**
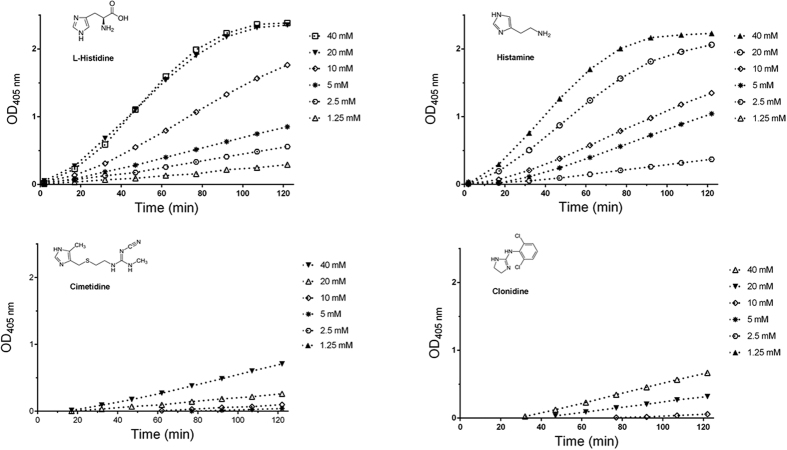
Cholinesterase-like activity of imidazole-bearing molecules. Progress curves of TNB^2−^ formation (pH 7.4, T 37 °C) ini the presence of a fixed [ATCh] (1 mM) and increasing [L-histidine], [histamine], [cimetidine] or [clonidine].

While N-alkylation of imidazole led to a complete loss of catalytic activity, the presence of a C-4 methyl substituent decreases the catalytic efficiency of the imidazole ring, suggesting a possible negative mesomeric effect of the alkyl substituents on the resonance of the molecule. To test this hypothesis, we monitored the ATCh hydrolysis in the presence of L-histidine, histamine, clonidine and cimetidine (Fig. 6). Histamine and L-histidine had a behaviour similar to that of imidazole, while clonidine and cimetidine showed a very low catalytic activities.

## Discussion

In the present study, we demonstrate for the first time that imidazole as well as natural (i.e. histamine and L-histidine) and synthetic (i.e. cimetidine and clonidine) molecules containing an imidazole ring or imidazole-derived moieties can act as organocatalysts increasing the hydrolysis rate of ATCh and PTCh. Moreover, we point out some aspects on the reaction kinetics and provide evidence on the possible catalysis mechanism.

The imidazole concentration-dependent enhancement of ATCh and PTCh hydrolyses were characterized by a sigmoidal behaviour. Such a kinetic profile was observed even in experiments carried out at a fixed imidazole concentration in the presence of the substrate at different concentrations. Noteworthy, imidazole catalysis occurred with a lag time of seconds or minutes from the start of the reaction and the same kinetic behavior was observed when ATCh was incubated in the presence of the other compounds tested (i.e., L-histidine, histamine, cimetidine and clonidine).

Sigmoidal behavior of progress curves are common in enzyme allosterism and in the cooperative binding of molecular oxygen to hemoglobins as a consequence of conformational changes of macromolecules, while it is uncommon for monomeric molecules. For instance, it has been described for autocatalytic events, where a product or an intermediate can also function as a catalyst, such as the ozone hole depletion in troposphere^22^. Factors related to the conditions of the two consecutive reactions involved in Ellman’s assay might be responsible for the unexpected kinetic behavior observed in the present study (please refer to specific discussion in Supplementary info section).

In our experimental model, the apparent *k*_*cat*_ of thioester hydrolysis in the presence of imidazole is greater for ATCh than PTCh, suggesting a positive mesomeric effect of the alkyl chain of the acidic moiety of the substrate on the electrophile carbonyl group of the thioester. This hypothesis is in agreement with previous findings on imidazole catalysis of other substrates^13^,^14^,^15^,^16^,^17^,^18^,^19^.

The efficiency of imidazole catalysis in ATCh hydrolysis increases as the solution becomes alkaline, suggesting a possible role of the nucleophile N-3 lone pair in the catalytic mechanism. Imidazole indeed is a highly polar, planar 5-membered ring aromatic compound characterized by the presence of a sextet of π-electrons. It exists in two equivalent tautomeric forms, protonated on each of the two nitrogen atoms, with p*K*_a_ values of 14.5, for the acidic N-1 proton, and 6.9 for the conjugated acid of the basic site N-3^23^. According to the Henderson-Hasselbalch equation, in diluted solutions, a Δ_pH(solution)-pKa(N-3)_ = 1 implies a 10:1 molar ratio between the imidazole molecules with N-3 atoms bearing a lone pair available to react as a Lewis base and molecules in the form of imidazolium cations.

The role of the nucleophile N-3 lone pair in the catalytic mechanism is further supported by the fact that N-alkylation of imidazole (as in 1-ethyl-3-methylimidazolium) led to a complete loss of activity. The methyl substituent on the imidazole C-4 position reduced catalytic activity suggesting a negative mesomeric effect of alkyl substituents on the aromatic cloud of the imidazole ring particularly on the ability of the N-3 lone pair to act as a Lewis base reactant. In line with this notion, elongation of the alkyl chain (as in L-histidine and histamine) causes a further decrease of the catalytic efficiency. On the other hand, adjacent substituents in the C4 and C5 positions (as in cimetidine) as well as the presence of C-2 substituents (as in clonidine) cause a marked reduction of catalytic efficiency, most probably due to a lost of aromaticity.

Finding of the present study also suggests a potential *in vivo* effect of imidazole-containing molecules. Organocatalysis has been recognized as an innovative method for the synthesis of natural and pharmaceutical products^24^ but little attention has been paid to its potential role in the *in vivo* processes. The high efficiency of macromolecular catalysts seems indeed to rule out the possibility of molecules with low catalytic efficiency to affect living systems. However, it should be pointed out that the actual concentration *in vivo* of the product of a catalyzed reaction reflects the balance between the rate of its synthesis and the rate of its disruption (steady-state condition). Therefore, even molecules with a low but persistent catalytic activity might alter this dynamic equilibrium and call for an homeostatic response.

The cholinergic system represents a different and even more complex extracellular condition. Beside its widely recognized role as a neurotransmitter of the central and peripheral nervous systems, acetylcholine (Ach) also represents a ubiquitous cell signaling molecule and an autocrine, juxtacrine and paracrine hormone. A non-neuronal cholinergic system has also been identified in the epithelial, endothelial and mesothelial cells, muscle fibers, parenchyma, and immune cells in human organisms. Ach plays a regulatory effect on many biological events including cell cycle, growth, survival, proliferation and differentiation, apoptosis, cell-to-cell contact, adhesion, organization of the cytoskeleton in cells and tissues of neuronal and non-neuronal types, cell secretion and resorption, trophism and even immune function and response to stress^25^,^26^,^27^,^28^,^29^,^30^.

As a consequence of this, it is not surprising that cholinergic status alterations have been found in several chronic diseases, drug addiction (e.g., alcohol, opiate, cocaine, ephedron, methylenedioxymethamphetamine, nicotine) and addiction-related serious complications, autistic spectrum disorders, malnutrition, unhealthy life styles (long-term high sugar and high fat intake, substance abuse) and cancer^31^. Noteworthy, low cholinesterase levels in serum and lymphocytes have been found in a considerably large number of patients with cancer, where low serum cholinesterase levels correlates with the extent of malignancy and histological classification^31^,^32^. Many identified risk factors for cancer initiation, development, and metastasis are also high-risk factors for cholinesterases^30^,^32^,^33^,^34^,^35^,^36^,^37^. On the other hand, cholinesterase activity can be injured by many physical and chemical agents such as radiations^38^, pesticides, nerve gas agents, pharmaceuticals^39^,^40^ and natural compounds^41^.

As for any other molecule within the body, the local steady state concentration of Ach results from the balance between the rate of secretion by cells and tissues and the rate of hydrolysis by cholinesterase isozymes^42^. The enzymes responsible for Ach clearance are detectable as membrane-bound molecules exposing their active site at the cell surface, in intracellular membranous organelles and as soluble forms in extracellular spaces, matrices and body fluids. Understanding better their regulation may be an important step for discovering new pharmacological strategies aimed at treating diseases based on defects in the cholinergic system.

Findings of the present study showing the ability of small molecules (such as histamine and histidine) to affect Ach metabolism provide new insights into the role of imidazole-based compounds in physiological and pathological processes.

As a matter of fact, several alimentary sources of histamine have been already identified, including many fishery products^43^. Moreover, histamine is present in almost all mammalian tissues, particularly in mastocytes, enterochromaffin-like cells of the oxyntic mucosa of the stomach, neurons of the central nervous system and blood cells^44^. Millimolar concentrations of histamine can be reached in inflammatory districts^45^,^46^ and probably in the gastrointestinal tract after ingestion of histamine rich-foods or foods triggering histamine release from the body. Although interactions between histamine and Ach have been already described both at peripheral and central levels^47^,^48^,^49^, findings of the present study may offer a novel point of view for the comprehension of the complex relationship between histaminergic and cholinergic systems. Noteworthy, while histamine metabolism leads to the loss of its biological activity, it does not alter the catalytic potential of its imidazole ring, at least until the complete removal from the internal milieu or the cell cytoplasm. Indeed, histamine undergoes an extracellular oxidative deamination of the primary amino group to imidazole acetaldehyde by diamine oxidase, or an intracellular methylation by histamine-*N*-methyltransferase, which is then oxidatively deaminated by monoamine oxidase B or diamine oxidase to yield *N*-methyl-imidazole acetaldehyde. Both aldehydes can finally be oxidized to form the corresponding acetates by aldehyde dehydrogenase^50^.

The relevance of the histamine precursor, histidine, is not restricted to the well-known role in a catalytic triad, such as the Ser-His-Glu in the acetylcholinesterase enzyme itself^51^. Histidine-containing dipeptides catalyse RNA formation in eutectic water^11^ and they are present at micromolar to millimolar concentrations in many tissues of vertebrates. Some of them, such as carnosine, may act as non-mast cell reservoir of histidine and histamine^52^. Noteworthy, daily intakes of histidine trough histidine rich foods, including beef, lamb, cheese, pork, chicken, turkey, soy, fish, nuts, seeds, eggs, beans, and whole grains, exceed the Reference Daily Intake in developed countries^53^. Therefore, understanding the effect on the cholinergic system induced by endogenous molecules and drugs containing an imidazole ring could be important also from a nutraceutical point of view.

## Conclusions

In conclusion, the present study suggests that millimolar concentrations of imidazole-based monomeric catalysts may potentially interfere with the cholinergic system supporting the role of histidine, histidine peptides, their derivatives and other autacoids as organocatalysts with potential application in the pharmaceutical and nutraceutical industry.

## Methods

### Chemicals and reagents

The following chemicals were obtained from Sigma and stored appropriately until use: imidazole, 4-methylimidazole, 1-ethyl-3-methylimidazolium chloride, histamine dihydrochloride (2-(4-Imidazolyl)ethylamine dihydrochloride), L-histidine, clonidine hydrochloride (2-(2,6-Dichloroanilino)-2-imidazoline hydrochloride), acetylthiocholine chloride, propionylthiocholine chloride, 5,5′-dithiobis(2-nitrobenzoic) acid (Ellman’s Reagent, DTNB), *Electrophorus Electricus* acetylcholinesterase.

### Cholinesterase-like activity assay

The reaction kinetics was firstly evaluated by curves obtained maintaining constant the substrates’ concentration (1 mM) and changing the concentrations of the organocatalyst tested (1.25–40 mM) or maintaining constant the organocatalyst concentration (1 mM) and changing the concentrations of substrates (1–32 mM). DTNB concentration was set at 200 μM (that is a quarter of the minimal substrate concentration used) to obtain the maximum expected absorbance for complete mixed disulfide and TNB^2−^ formation. This consideration was done assuming a TNB^2−^ molar absorption coefficient at 405 nm of 1.37 × 10^4^ M^−1^ cm^−1^ at 37 °C, that is, an absorbance value well within the sensitivity range of the spectrophotometer. The same TNB^2−^ ε_0_ was assumed in calculations. Positive control was obtained by adding acetylcholinesterase to the reaction mixture. Cholinesterase-like activity was tested in 96 well plates using the Ellman’s assay^21^, slightly modified. All reagents were dissolved in phosphate-pyrophosphate buffer (50 mM), prepared by titrating sodium acid pyrophosphate (Na_2_H_2_P_2_O_7_) with sodium orthophosphate (Na_3_PO_4_) to pH 7.4, except for assessment of the effect of pH on catalysis. This was assessed in the range of 5.8–8.0 by adding 1 mM ATCh to a 50 mM buffered solution containing 5 mM imidazole and 200 μM DTNB.

To measure the concentration dependency of imidazole cholinesterase-like activity, serial 1:2 dilutions of 50 μL of 4X imidazole (i.e. 160 mM) were performed from column 12 to 1 in duplicate; the reaction mixture was completed by adding 50 μL of 4X ATCh (i.e. 4 mM) and 100 μL of 2X DTNB (i.e. 400 μM).

The progress curves of TNB^2−^ formation in the presence of a fixed [imidazole] and increasing [ATCh] or [PTCh] were obtained by serially diluting 1:2 50 μL of 4X [ATCh] or [PTCh] (i.e. 128 mM) from column 12 to 1 in duplicate; the reaction mixture was completed by adding 50 μL of 4X imidazole (i.e. 4 mM) and 100 μL of 2X DTNB (i.e. 400 μM).

The pH influence on imidazole organocatalysis was assessed by mixing 50 μL of 4X [ATCh] (i.e. 4 mM), 50 μL of 4X [imidazole] (i.e. 20 mM) and 100 μL of 2X DTNB (i.e. 400 μM), each dissolved in the appropriate buffer (i.e. phosphate-pyrophosphate buffer (50 mM) titrated to different pH in the range of 5.8–8.0 with sodium orthophosphate and dispensed in triplicate from column 1 to 12.

Comparison of 1-ethyl-3-methylimidazolium and 4-methylimidazole vs. imidazole activity was measured by mixing 50 μL of 4X [ATCh] (i.e. 4 mM), 50 μL of 4X DTNB (i.e. 800 μM) and 100 μL of 2X candidate catalyst (i.e. 80 mM) in triplicate, from column 1 to 9.

The cholinesterase-like activity of imidazole-bearing molecules was tested by serially diluting 1:2 50 μL of 4X [candidate catalyst] (i.e. 160 mM) from column 12 to 1 in duplicate and adding 50 μL of 4X ATCh (i.e. 4 mM) and 100 μL of 2X DTNB (i.e. 400 μM).

Controls for blank hydrolysis were included in every assay. The spontaneous hydrolysis of choline thioesters in aqueous solutions was measured by avoiding the addition of catalyst in rows G and H. The additional hydrolysis coming from the imidazole-induced disruption of DTNB (see results) was measured by avoiding choline thioester addition in rows E and F. A final volume of 200 μL was maintained by adding the corresponding volume of buffer solution.

Immediately after completion of sample preparation, plates were incubated at 37 °C and briefly shaken just before recording their absorbance at 405 nm (due to DTNB-derived yellow product, TNB^2−^, Fig. 1) by Infinite 200 PRO NanoQuant instrument (Tecan Italia Srl, Milan, Italy), 2 min after the reaction mixture set up and then at 15 min intervals.

The blank hydrolysis noise has been subtracted from the recorded absorbance values and data submitted to numerical analysis.

### Statistical analysis

Data were analyzed by appropriate statistics (i.e. cubic spline interpolation, Fisher-Z statistics, linear and non-linear regression) by using the Prism 4.0 software (GraphPad Software, Inc., La Jolla, USA). P < 0.01 was adopted as limit of significance.

## Additional Information

**How to cite this article:** Nieri, P. *et al*. Cholinesterase-like organocatalysis by imidazole and imidazole-bearing molecules. *Sci. Rep.* **7**, 45760; doi: 10.1038/srep45760 (2017).

**Publisher's note:** Springer Nature remains neutral with regard to jurisdictional claims in published maps and institutional affiliations.

## Supplementary Material

Supplementary Information
