# Supplementary material for: Cholinesterase-like organocatalysis by imidazole and imidazole-bearing molecules
Source: Sci Rep. 2017 Apr 3;7:45760. doi: 10.1038/srep45760 (PMC5377937; doi:10.1038/srep45760)
Supplement: Supplementary Information [file srep45760-s1.pdf]

Cholinesterase-like organocatalysis by imidazole and imidazole-bearing molecules

Paola Nieri, Sara Carpi , Stefano Fogli , Beatrice Polini, Maria Cristina Breschi & Adriano Podestà

In Ellman's assay, the following, two consecutive reactions are involved:

$$RS\text{-}CO\text{-}R' + H_2O \overset{k_1}{\text{---->}} RSH + R'COOH + DTNB \underset{k_3}{\overset{k_2}{<==>}} RSSR'' + TNB^{2-} + R'COO^- \quad (\mathbf{1})$$

In the experimental condition adopted in the current study (i.e., no molar excess of DTNB), TNB^{2-} formation occurred with a second order kinetics and the reverse reaction to DTNB was not negligible. On the other hand, the thioester hydrolysis can be regarded *per se* as irreversible, with a pseudo first order kinetics ($v=k[\text{thioester}]^1[H_2O]^0$) due to the molar excess of H_2O. At the same time, it is the rate-limiting step of the complete reaction, as revealed by the ~10,000 fold increase in velocity of TNB^{2-} formation occurring when acetylcholinesterase instead imidazole is added to the thioester substrate solution.

Taking into account the abovementioned considerations, a rational for the observed sigmoid shape of the progress curves can be proposed. Due to spontaneous hydrolysis of thioester molecules, the second reaction is at a $K_{eq}= k_{2/}\, k_3>1$ equilibrium, responsible for a non-zero absorbance (vs. the solvent of solution) already before the organocatalyst addition. Since catalyst addition, the thioester decreases at its highest $v=k_{1cat}[\text{thioester}]^1[H_2O]^0$, being maximal the [thioester]. Nevertheless, two factors will contribute to maintain unchanged the sample absorbance: i) the slow $v=k_{1cat}[\text{thioester}]^1[H_2O]^0$ as compared to the fast $k_{2/}\, k_3$=K>1 equilibrium; ii) the quantitative difference between the number of new RSH molecules entering the solution and that of RSH molecules already in solution as a consequence of the chemical equilibrium. An induction time will be thus observed until the $\Delta[RSH]_{cat}$ becomes high enough to make the $k_2[RSH]^1[DTNB]^1>$ $k_3[RSSR'']^1[TNB^{2-}]^1$. Obviously, the lower the k_{1cat} value (depending on the catalyst in use), the greater the induction time. Similarly, the higher the [thioester] or the [Cat], the shorter the induction time. Finally, the more intact the substrate, the more sensitive the system and shorter the induction time.

On the other hand, being the first reaction of (**1**) the rate-limiting step due to the low value of k_{1cat}, the maximal value of $\Delta v=(k_2[\mathrm{RSH}]^1[\mathrm{DTNB}]^1- k_3[\mathrm{RSSR''}]^1[\mathrm{TNB}^{2-}]^1$ cannot be greater than $v=k[\mathrm{thioester}]^1[H_2O]^0$. In other words, the velocity of TNB^{2-} formation cannot be greater than the velocity of [RSH] restoring as substrate of the second reaction in (**1**). This implies that the slope of the linear tract of the sigmoid can be assumed as the apparent k_{cat} of the first reaction, i.e. the apparent rate constant of the catalyzed reaction. Since then the thioester, RSH and TNB^{2-} consumption will cause the Δv to progressively decrease up the 0 value of equilibrium, as evident in the second half of the progress curve. In this study, linear regions (identified through the Fisher-Z statistics) of the progress curves have then been taken into account for initial velocity (v_0) and apparent k_{cat} assessment. At the same time, the apparent k_{cat} values are underestimated in respect to true k_{cat}, due to the loss of substrate that occurs during the latency phase of the progress curve. This implies that apparent k_{cat} values are meaning only in relative terms, when results of experiments performed with the same [Cat] and [thioester] are compared.
